# High risk factors, molecular features and clinical management for radioactive iodine-refractory differentiated thyroid carcinoma

**DOI:** 10.3389/fonc.2025.1644562

**Published:** 2025-08-25

**Authors:** Tengyun Ma, Yiting Xie, Xinyi Long, Feng Ye

**Affiliations:** Department of Pathology, Institute of Clinical Pathology, Frontiers Science Center for Disease-related Molecular Network, West China Hospital, Sichuan University, Chengdu, Sichuan, China

**Keywords:** radioactive iodine-refractory differentiated thyroid carcinoma (RAIR-DTC), sodium-iodide symporter (NIS), high-risk factors, molecular pathogenesis, clinical management

## Abstract

Despite the generally favorable prognosis of differentiated thyroid carcinoma (DTC) following surgery and radioactive iodine (RAI) therapy, approximately 10% of cases eventually develop resistance to RAI. This condition, known as radioiodine-refractory differentiated thyroid carcinoma (RAIR-DTC), is associated with a poor prognosis, with a 10-year survival rate of only 10% from the time of metastasis detection. The limited availability of safe and effective alternative treatments poses a significant challenge to clinical management. However, early identification and intervention targeting high-risk factors are critical for preventing disease progression. Integrating current insights into DTC pathogenesis with established clinical strategies offers valuable opportunities to inform the development of novel therapies and improve patient outcomes. Hence, in this review, we first examine high-risk predictors of RAIR, including demographic factors (e.g., age, sex), gene mutations (e.g., RAS, BRAF, TERT), high-risk histopathological subtypes (e.g., extrathyroidal extension and the tall cell variant), and serum biomarkers (e.g., thyroglobulin and Cyfra 21.1), all of which are widely recognized for monitoring and risk stratification. Notably, we also emphasize that inappropriate pharmacological management of comorbidities—such as diabetes, myeloid leukemia, and hypertension—may suppress sodium-iodide symporter (NIS) expression and RAI uptake, thereby contributing to RAIR development. We then summarize the molecular mechanisms underlying impaired NIS expression and function in RAIR-DTC, followed by a discussion of recent advances in clinical treatment, focusing on the efficacy and safety of both approved and investigational therapeutic agents.

## 1 Introduction

Thyroid carcinoma (TC) is the most common malignancy of the endocrine system and is categorized into three main histological subtypes: differentiated thyroid carcinoma (DTC), which includes both well-differentiated and poorly differentiated forms (PDTC); undifferentiated carcinoma, known as anaplastic thyroid carcinoma (ATC); and medullary thyroid carcinoma, which arises from calcitonin-producing C-cells. Among these, DTC is the most prevalent, accounting for over 90% of all TC cases (1, 2), and comprises papillary thyroid carcinoma (PTC), follicular thyroid carcinoma (FTC), and Hürthle cell carcinoma (3). Epidemiological data indicate a sharp increase in TC incidence in China, with more than 460,000 new cases reported annually, approximately 120,000 in males and 340,000 in females, making it the third most common cancer in the country and responsible for around 11,500 deaths each year (4). Following initial treatment, which typically includes thyroidectomy, thyroid-stimulating hormone (TSH) suppression, and radioactive iodine (RAI) therapy (5), DTC generally carries a favorable prognosis (6). Nonetheless, distant metastases develop in approximately 7–23% of DTC patients (7), and nearly two-thirds of these cases undergo dedifferentiation, leading to reduced iodine uptake and the development of RAI resistance (8), known as radioactive iodine-refractory differentiated thyroid carcinoma (RAIR-DTC) (9). RAIR-DTC is defined by the following criteria (1): no RAI uptake in known DTC lesions; or (2) disease progression within one year following RAI therapy despite iodine exposure. Disease progression may manifest as:① increased serum thyroglobulin (TG) or TG antibodies, lesion enlargement, or the appearance of new lesions; ②worsening symptoms related to the original disease, emergence of new symptoms, or death (10). Clinically, RAIR-DTC is typically classified into two phenotypes: asymptomatic indolent and symptomatic progressive. The asymptomatic form is characterized by low tumor burden, absence of significant symptoms, and stable or slowly progressing disease. The symptomatic form can be further divided into oligometastatic and advanced or widely metastatic subtypes (11).

Conventional therapies for RAIR-DTC, such as cytotoxic agents like doxorubicin, used alone or in combination with cisplatin or interferon alpha-2b, have demonstrated low response rates and are often accompanied by severe adverse effects (AEs) (12, 13). Advances in understanding the molecular mechanisms of RAIR-DTC have led to the development of targeted therapies, which have significantly extended progression-free survival (PFS). Nevertheless, only Apatinib and Lenvatinib (specifically in patients over 65 years of age) have shown a survival benefit in terms of overall survival (OS) (14, 15). Despite these benefits, targeted therapies are associated with a range of AEs, including hypocalcemia (16), salivary gland dysfunction (17), and metastatic diseases (lung, bone, liver and brain metastases) (18–20), as well as thyroid hormone imbalance (21) and cardiovascular diseases (hypertension, diabetes, obesity, dyslipidemia, heart rhythm abnormalities and heart failure) (22). Currently, there is also no consensus on the optimal timing for initiating multi-kinase inhibitors (MKIs) therapy in asymptomatic RAIR-DTC patients. Delaying treatment risks disease progression and loss of control, while premature initiation may negatively impact quality of life (23). Given that RAIR-DTC often results from thyroid dedifferentiation, redifferentiation therapy aimed at restoring RAI uptake offers a potential treatment avenue. However, most redifferentiation agents remain in experimental stages or have shown limited clinical efficacy (24, 25). Notably, whether through emerging targeted therapies or redifferentiation strategies, tumor cells frequently develop resistance via new genetic mutations, compensatory activation of bypass pathways, or alterations in drug targets (26, 27). Due to the lack of safe, effective and specifical therapies for RAIR-DTCs, the prognosis remains poor, with a 5-year disease-specific survival rate of 60–70% and a 10-year overall survival rate of merely 10% following metastatic progression (28).

Given the limited availability of specialized therapies for RAIR-DTC, numerous studies have aimed to identify predictors of its development. In this review, we examine common high-risk factors, including demographic character (e.g., age, ethnicity), genetic mutations (e.g., RAS, BRAF, TERT promoter, and RET), clinicopathological features (e.g., hobnail variant PTC, and lymph node metastasis), and early monitoring markers (Thyroglobulin (TG), Cyfra 21.1), that contribute to RAIR-DTC risk stratification. We also highlight potential inducing factors that may promote RAIR-DTC onset, such as comorbidities (e.g., diabetes and myeloid leukemia) and inappropriate use of certain medications (e.g., metformin, nilotinib, and etilefrine), which may impair RAI uptake and facilitate disease progression. Additionally, we summarize the molecular basis of RAIR-DTC, primarily characterized by reduced expression and impaired function of the sodium-iodide symporter (NIS). This impairment is often driven by genetic alterations (e.g., RAS, RAF, PIK3CA) that activate signaling pathways such as MAPK, PI3K/Akt, and AMPK. Finally, we discuss recent advances in clinical management, including approved therapies and agents currently under investigation in clinical trials. Moving forward, research should prioritize early identification of high-risk factors to prevent disease progression. At the same time, studies on molecular pathogenesis and treatment strategies should evaluate their influence on NIS expression and function, with the goal of minimizing AEs and improving patient outcomes.

## 2 High-risk factors for RAIR-DTCs

**Figure 1:**
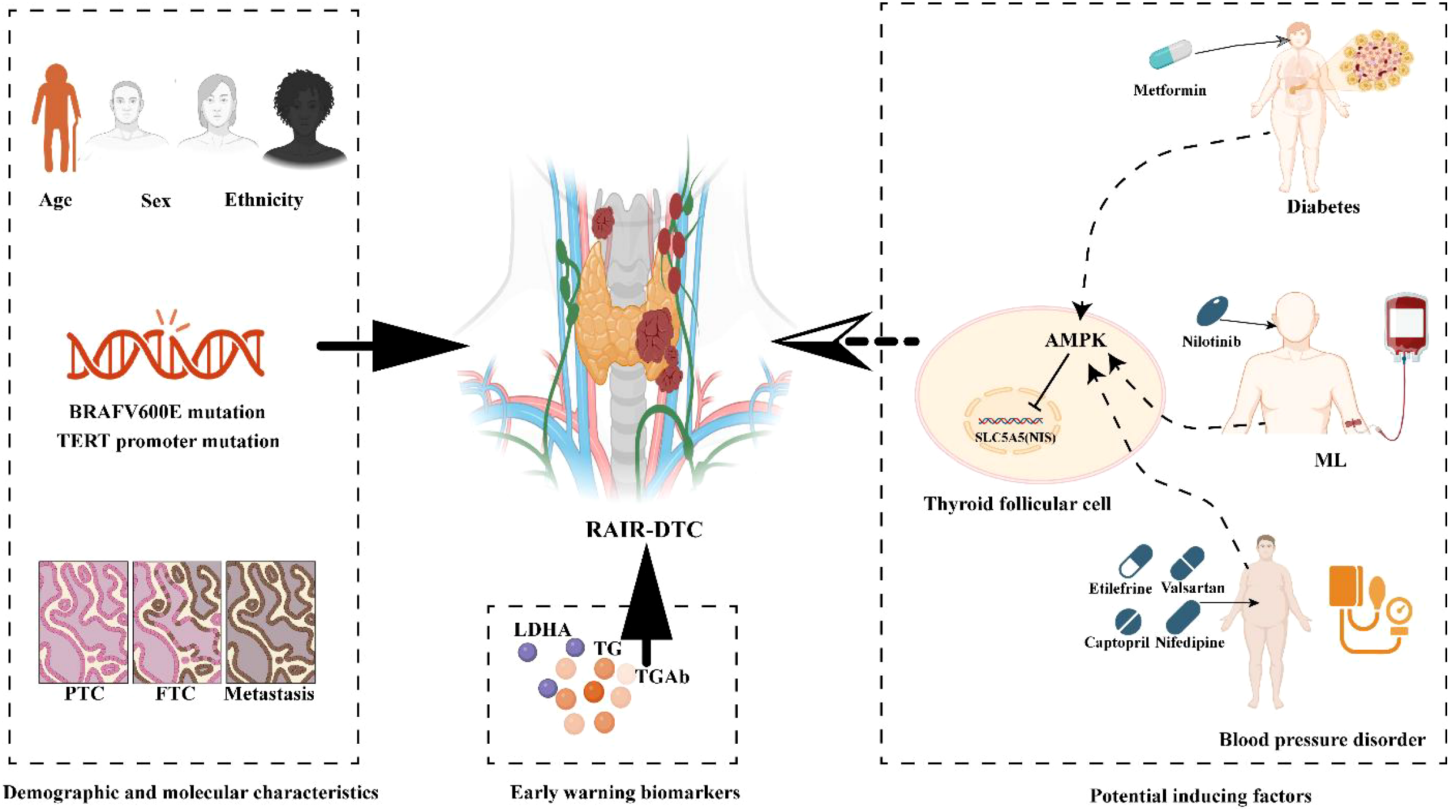
Schematics showing active preventive strategies of RAIR-DTC. Common high-risk factors include demographic characteristics, genetic mutations, clinicopathological features, early warning markers, and promoting factors related to comorbidities and their associated medications. Demographic characteristics include patient age, gender, and ethnicity. Genetic mutations include common BRAF V600E and TERT promoter mutations. Clinicopathological features include Tall cell variant and Lymph node metastasis. Common monitor biomarkers include TG and TGAb. Potential inducing factors in unsuitable combination led by related comorbidity. TG, Thyroglobulin; TGAb, Thyroglobulin Antibodies; LDHA, Lactate Dehydrogenase A; ML, Myeloid leukemia. The diagram was created with BioRender.com.

Accurate identification of high-risk factors for RAIR-DTC is essential for guiding personalized prevention and treatment strategies. In this context, we conducted a retrospective literature review to synthesize key risk factors associated with RAIR-DTC development. This comprehensive analysis includes demographic characteristics, genetic mutations, clinicopathological features, early monitoring markers, and promoting factors related to comorbidities and their associated medications (29–32) (**Figure 1**).

### 2.1 Demographic characteristics

Common demographic factors include age, ethnicity, sex, geographic distribution, and lifestyle (33). In this section, we review the relevant literature and summarize the associations between age, gender, ethnicity, and lifestyle and the development of RAIR-DTC (**Table 1**).

**Table 1:** Summary of clinical and lifestyle factors associated with RAIR-DTC.

Category	Research findings	Ref.
Age	Individuals aged >45 or >55 years have an increased risk of RAIR-DTC;	(29, 34, 35)
Age	Individuals age cut-offs of 45 and 75 years were predictive for OS but not for PFS;	(36)
Sex	The proportion of RAIR is higher in males than in females (P<0.05); some studies have also found a higher proportion in females than males, but without statistical significance;	(37, 38)
Ethnicity	White individuals have a lower risk of RAIR; Black individuals have an increased risk of RAIR; specific genotypes (TG/BRCA1/NSMCE2) are associated with Black individuals	(34, 39)
Life style	Smoking and BMI ≥24 kg/m² are associated with an increased incidence of RAIR	(29)

#### 2.1.1 Age and sex

Numerous studies have identified age threshold as a predictive factor for RAIR-DTC. Retrospective analyses by Shobab et al. and Chen et al. found that patients over 45 years old had a higher odds ratio (OR) for developing RAIR-DTC (34, 35). Similarly, Li et al. reported an increased incidence of RAIR in patients over 55 years of age (29). Interestingly, Saie et al. noted that age thresholds of 45 and 75 years were predictive of OS, but not PFS, in RAIR-DTC patients (36). Regarding sex, Wang et al. observed a significantly higher proportion of male RAIR patients compared to females (*P* < 0.05), with logistic regression confirming male sex as a predictive factor (37). In contrast, Liu et al. found a higher proportion of female RAIR patients, although the difference was not statistically significant (38).

#### 2.1.2 Ethnicity and life style

Shobab et al. analyzed the relationship between ethnicity and RAIR-DTC, finding that individuals of white ethnicity had a lower OR for RAIR, whereas those of black ethnicity had a higher OR (34). In a germline genetic analysis comparing African American and Caucasian patients, Hurst et al. identified specific haplotypes—TG, BRCA1, and NSMCE2—that were associated with RAIR occurrence in African American patients with over 80% African ancestry (39). Regarding lifestyle factors, Li et al. reported that smoking and a body mass index (BMI) of 24 kg/m² or higher were associated with an increased risk of RAIR development (29).

### 2.2 Gene alterations

The Fifth Edition of the World Health Organization (WHO) Classification of Endocrine and Neuroendocrine Tumors highlights genetic mutations—particularly BRAF—as key early drivers in the development of RAIR-DTC and valuable predictors of its occurrence (3). For instance, in a molecular analysis of 220 cases of distantly metastatic DTC, Mu et al. compared non-RAIR (N = 81) and RAIR (N = 139) cohorts. Their results showed significantly higher mutation frequencies in the RAIR group for BRAF^V600E^ (59.7% vs. 17.3%), TERT promoter (43.9% vs. 7.4%), and TP53 (43.9% vs. 7.4%). In contrast, rearranged during transfection (RET) mutations were less common in RAIR-DTCs than in non-RAIRs (15.8% vs. 39.5%) (32). In this section, we emphasize the associations between RAS, BRAF^V600E^, TERT, RET, and NTRK mutations and RAIR prediction, underscoring their relevance for prognosis and therapeutic decision-making in RAIR-DTC (**Table 2**).

**Table 2:** Gene alterations in RAIR-DTC and clinical implications.

Molecular Alteration	Prevalence/Subtypes	Functional Consequences	Clinical Associations	Ref.
RAS family mutations	Overall: 35.4% in TC NRAS: 69.47% HRAS: 25.83% KRAS: 6.92%	Differentiation markers (TG, TPO, TSHR)↓; Cell proliferation↑; Chromosomal instability↑; Requires co-mutations for full malignancy	Aggressive phenotype when co-mutated with TERT; Mortality risk↑	(40–43)
BRAF^V600E^	Predominant in PTC	NIS expression↓;Cell proliferation and oncogenesis↑	Tumor size, lymphovascular invasion, lymph node metastasis↑; Independent predictor of RAIR-DTC	(32, 35, 45, 46)
TERT promoter mutations	Frequent in DTCs	Telomerase activation; Cellular immortalization; Dedifferentiation induction	Stronger association with RAI avidity loss than BRAF; Accelerates RAIR progression; Major genetic driver of RAIR	(30, 48–50)
RET rearrangements	~20% of PTCs	Dedifferentiation of DTCs; Impairs RAI uptake/metabolism	Higher clinical significance for RAIR development in pediatric TC	(51, 52)
NTRK fusions	Common subtypes: ETV6-NTRK3 (64.4%) TPM3-NTRK1 (8.4%) SQSTM1-NTRK3 (6.8%)	Oncogenic transformation; Promotes aggressive histology	High-grade features (mitosis, LVI, ETE)↑; Distant lymph node metastasis↑; Diagnostic/prognostic markers	(54, 55)

↑, upgrade; ↓, downgrade.

#### 2.2.1 RAS

The RAS gene family (NRAS, HRAS, KRAS) encodes GTP-binding proteins, and activating mutations in these genes are frequently observed in TCs, with an overall prevalence of approximately 35.4%—NRAS accounting for 69.47%, HRAS for 25.83%, and KRAS for 6.92% (40). Studies by Bond et al., Monaco et al., and Portella et al. have shown that RAS mutations reduce the expression of thyroid differentiation markers such as thyroglobulin (TG), thyroid peroxidase (TPO), and the thyrotropin receptor (TSHR), while promoting increased thyroid cell proliferation. Although RAS mutations alone are insufficient to drive full malignant transformation, the presence of co-occurring mutations, particularly TERT promoter mutations, has been linked to more aggressive tumor phenotypes and increased mortality in RAS-mutant DTCs (41, 42). Furthermore, Saavedra et al. demonstrated that RAS mutations promote chromosomal instability in PCCL3 cells, potentially facilitating malignant progression by increasing susceptibility to additional genetic alterations (43).

#### 2.2.2 BRAFV600E

BRAF, a member of the RAF kinase family, plays a key role in cell signaling pathways that regulate proliferation and survival. The BRAF^V600E^ mutation, which is more prevalent in PTCs than RAS mutations, drives oncogenesis by promoting uncontrolled cell proliferation (44). Dong et al. demonstrated that BRAF^V600E^ significantly reduced sodium-iodide symporter (NIS) expression in a subset of PTC patients without Hashimoto’s thyroiditis (*P* = 0.046) (45). Additionally, Makboul et al., in an analysis of clinical data from 78 PTC patients, found that BRAF^V600E^ was significantly associated with increased tumor size, lymphovascular invasion, and lymph node metastasis (46). Furthermore, both Mu et al. and Wang et al. identified BRAF^V600E^ as an independent predictor of RAIR-DTC (32, 35).

#### 2.2.3 TERT promoter

The TERT gene encodes the catalytic subunit of telomerase, an enzyme critical for maintaining telomere length and promoting cellular immortality. Mutations in the TERT promoter are commonly observed in DTC and are strongly associated with tumor dedifferentiation (47). Yang et al. conducted a retrospective analysis of 66 DTC cases with distant metastases and found that TERT promoter mutations were more strongly associated with reduced RAI avidity and negative RAI uptake compared to BRAF^V600E^ mutations (48). Similarly, Tan et al., in a cohort of 243 patients (non-RAIR: N = 212; RAIR: N = 31), reported that TERT promoter mutations accelerated progression to RAIR (49). Parvathareddy et al. also demonstrated a significant association between TERT promoter mutations and RAIR-DTC through multivariate analysis of 268 RAIR-DTC cases (30). Furthermore, Ju et al., using next-generation sequencing on 278 RAIR-DTC tumor samples, confirmed that TERT promoter mutations were the predominant genetic drivers of RAIR in adults (50).

#### 2.2.4 RET

The RET proto-oncogene, located at chromosome 10q11.2, encodes a receptor tyrosine kinase involved in several TC subtypes. RET rearrangements, present in approximately 20% of PTCs, promote dedifferentiation of DTCs and significantly impair RAI uptake and metabolism (51). Notably, thyroid cells in children are more vulnerable to ionizing radiation and have reduced DNA repair capacity, making them more susceptible to RET rearrangements and subsequent malignant transformation (52). These rearrangements play a critical role in compromising RAI responsiveness and contribute to the development of RAIR-DTC. Therefore, detecting RET mutations and fusions in pediatric TC cases holds important clinical significance.

#### 2.2.5 NTRK

The TRK family of transmembrane receptorsranes. TRKB, and TRKC,to encoded by the NTRK1, NTRK2, and NTRK3 genes, respectively. NTRK gene fusions have oncogenic potential in both neural and non-neural tissues (53). In DTCs, the most common fusion types include ETV6udeEN. (64.4%), TPM34%),N. (8.4%), and SQSTM1,N.CI (6.8%) (54). Notably, non-secretory TCs characterized by multinodular growth of eosinophilic cells harboring NTRK fusions are often associated with more aggressive clinical behavior. Chu et al. reported that NTRK fusion–positive. PTCs frequently exhibit high-grade features such as elevated mitotic activity, extensive lymphatic invasion, and extrathyroidal extension (ETE) (55). Similarly, Pekova et al. found that thyroid cancers with NTRK1 fusions show increased lymphatic invasion and distant lymph node metastases (54). Both NTRK1 and NTRK3 fusions have emerged as valuable diagnostic and prognostic biomarkers in TC.

### 2.3 Clinical-pathological features

Although no definitive consensus exists regarding the histopathological features that define RAIR-DTC, several clinicopathological characteristics commonly observed in RAIR-DTC have been identified as independent predictors of its development (56). This section examines the role of high-risk histopathological subtypes and other clinicopathological factors in predicting RAIR-DTC. These insights are critical for advancing personalized precision medicine and improving prognostic assessment (**Table 3**).

**Table 3:** Clinicopathological characteristics and early monitoring markers predictive of RAIR-DTC.

Category	Specific factors	Key Findings & Significance	Ref.
Clinicopathological Predictors	High-Risk Histopathological Subtypes (e.g., ETE, Tall cell variant, Sclerosing diffuse, Hobnail variant PTC, FTC/Hürthle cell, PDTC)	Associated with RAIR incidence; serve as independent predictors for RAIR-DTC development	(29, 56)
	Other Clinicopathological Features (Lymph node metastasis Number ≥4, Lymph node metastasis Rate≥53%; Vascular Invasion; Synchronous Distant Metastases (cervical, pulmonary, bone) at diagnosis; Cervical/Pulmonary Recurrence)	Significantly and positively correlated with RAIR prevalence; identified as independent predictive factors	(29, 31)
Early Serum & Molecular Monitoring Markers	Thyroglobulin (TG) & Anti-TG Antibodies (TGAb)	Low post-I^131^ TG reduction (<25.3%), elevated preoperative TG, and low △s-TG/△s-TSH (<1.5) independently predict RAIR; TGAb testing is essential due to interference.	(58–60)
	TPO Expression	Absence of TPO expression in metastatic lesions predicts RAIR occurrence	(62)
	Cyfra 21.1 & LDHA	Elevated Cyfra 21.1 in RAIR vs. RAI-avid cases (*P*=0.047); LDHA levels inversely correlate with RAI uptake, suggesting utility in RAI resistance monitoring	(63, 64)
	Other biomarkers	miR-139-5p downregulation and AXL overexpression (associated with BRAF^V600E^ and NIS suppression) predict RAIR; high LDL-C/TC ratio correlates with RAIR incidence.	(65–67)

#### 2.3.1 High-risk histopathological subtypes

Li et al. and Luo et al. conducted retrospective analyses involving approximately 6,594 cases and found that several histopathological subtypes, such as extrathyroidal extension (ETE), tall cell variant, diffuse sclerosing and hobnail variants of PTC, follicular thyroid carcinoma (FTC, including Hürthle cell), and poorly differentiated thyroid carcinoma (PDTC), are significantly associated with RAIR-DTC. These features serve as independent predictors for the development of RAIR-DTC (29, 56).

#### 2.3.2 Other clinical-pathological features

Li et al. found that the number of lymph node metastases (≥4), lymph node metastasis rate (≥53%), and pN stage (N1) were significantly and positively associated with the prevalence of RAIR-DTC (29). In a comparative analysis of 159 RAIR cases and 759 non-RAIR cases, Schubert et al. identified vascular invasion, synchronous cervical, pulmonary, and bone metastases at initial diagnosis, as well as cervical and pulmonary recurrence during follow-up, as independent predictive factors for RAIR development (31).

### 2.4 Early monitoring markers for RAIR-DTCs

Currently, only a limited number of early monitoring markers for RAIR-DTC have been well established (57). Clinically significant biomarkers include thyroglobulin (TG), anti-thyroglobulin antibodies (TGAb), thyroid-stimulating hormone (TSH), as well as molecular markers such as microRNAs (miRNAs) and tyrosine kinase receptors. Notably, TG and TGAb have been incorporated into the 2025 edition of the China Guidelines for Integrated Treatment of Tumors as recommended serum markers for RAIR-DTC follow-up (10). In this section, we summarize several commonly used biomarkers for predicting RAIR occurrence (**Table 3**).

#### 2.4.1 Thyroglobulin

Thyroglobulin (TG) is an effective biomarker for monitoring clinical outcomes in patients with distant metastatic DTC. Sa et al. reported that a post-I¹³¹ TG reduction of less than 25.3% in these patients predicted a poor response to subsequent I¹³¹ therapy (58). In a retrospective analysis of 876 DTC cases (786 non-RAIR vs. 90 RAIR), Cheng et al. found that elevated preoperative TG (pre-TG) levels were significantly associated with RAIR development and served as an independent predictor (59). Meng et al. developed a nomogram model using a cutoff value of △s-TG/△s-TSH < 1.5(where △s-TG represents the difference between pre-treatment stimulated and suppressed TG, and △s-TSH denotes the difference in TSH levels before and after treatment) to predict RAIR-DTC progression (60). Importantly, serum TG measurements should be interpreted alongside anti-thyroglobulin antibody (TGAb) levels, as TGAb can interfere with TG accuracy.

#### 2.4.2 Thyroid peroxidase

Nilsson et al. demonstrated a positive correlation between thyroid peroxidase (TPO) expression and RAI avidity by analyzing surgical specimens, including primary tumors and lymph node metastases, from 28 DTC patients (61). Similarly, Zelinskaya et al., through immunocytochemical analysis of 104 metastatic DTC lesions, confirmed that the absence of TPO expression is a predictive marker for RAIR development (62).

#### 2.4.3 Cyfra 21.1 and lactate dehydrogenase A

Jeong et al. reported that significantly higher serum Cyfra 21.1 levels in patients with metastatic TCs compared to those with non-metastatic disease and healthy controls (distant metastasis: N = 51; non-distant metastasis: N = 76; *P* = 0.012). Among the metastatic group, RAIR patients (N = 13) exhibited significantly higher Cyfra 21.1 levels than RAI-avid patients (N = 12; *P* = 0.047), suggesting its potential as a biomarker for RAIR-DTC, particularly in cases where TG is undetectable (63). Additionally, Tian et al. demonstrated an inverse correlation between lactate dehydrogenase A (LDHA) expression and RAI uptake in a cohort of 69 DTC patients, indicating a potential role of LDHA in mediating RAI resistance (64).

#### 2.4.4 Others

Pecce et al. reported that miR-139-5p was significantly downregulated in RAIR-DTC patients (N = 14) compared to non-RAIR-DTC patients (N = 12). Overexpression of miR-139-5p enhanced NIS expression and RAI uptake, suggesting its potential as a predictive biomarker for RAIR-DTC (65). Additionally, Collina et al. found that a strong association between high expression of anexelekto (AXL), BRAF^V600E^ mutations, and RAIR occurrence (*P* < 0.0001) in a cohort of 110 PTC patients and 5 controls. Their findings indicate that elevated AXL expression may contribute to RAIR by suppressing NIS expression, thus serving as a potential predictive marker (66). Liu et al. also identified that a significant correlation between the low density lipoprotein-cholesterol-total cholesterol ratio (LDL-Ch/TCh) ratio and RAIR incidence in TC patients (RAIR: N = 12; controls: N = 24) (67).

### 2.5 Promoting factors from comorbidities and its medication

Research suggests that environmental factors, dietary habits, and metabolic syndrome, including central obesity and insulin resistance, may contribute to the onset and progression of RAIR-DTC (68–70). DTC is also commonly comorbid with conditions such as diabetes, myeloid leukemia (ML), and blood pressure disorders, which may influence disease progression, potentially through activation of the AMPK signaling pathway (71–75). Although limited studies have examined the impact of comorbidities and associated medications on RAIR development, our retrospective literature review highlights several medications linked to DTC related comorbidities that may act as potential inducers of RAIR, as summarized in **Table 4**.

**Table 4:** Comorbidity and unsuited drugs for contributing to RAIR-DTCs.

Comorbidity	Risk drugs	Risk effect	Mechanism	Ref
DTC and Diabetes	Metformin	Downregulation of NIS	Activating AMPK pathway	(84)
DTC and Chronic myeloid leukemia	Nilotinib	Downregulation of NIS	Activating AMPK pathway	(71)
DTC and hypotension	Etilefrine	Downregulation of NIS	Activating AMPK pathway	(101)
DTC and hypertension	Valsartan	Downregulation of NIS	Activating AMPK pathway	(97)
DTC and hypertension	Captopril	Downregulation of NIS	Activating AMPK pathway	(96)
DTC and hypertension	Nifedipine	Downregulation of NIS	Activating AMPK pathway	(102)

#### 2.5.1 Diabetes and RAIR-DTC

Studies have established a connection between diabetes mellitus and thyroid disorders, indicating that diabetes related traits such as elevated insulin levels, insulin resistance, metabolic dysregulation, and oxidative stress may exacerbate the severity of thyroid conditions (76–78). Zhang et al. utilized the TyG index, a marker of insulin resistance, to analyze a cohort of 47,710 subjects, revealing a significant increase in the prevalence of thyroid disorders and the risk of subclinical hypothyroidism associated with higher TyG index values (79). Moreover, impaired sensitivity to TSH or reduced TSH levels in individuals with diabetes contribute to metabolic dysregulation, resulting in decreased RAI sensitivity and diminished expression of NIS (80, 81). The oxidative stress induced by diabetes, characterized by elevated lipid peroxides and downregulated TSH levels, disrupts thyroide redox balance of the thyroid gland (81, 82). Azouzi et al. demonstrated that the upregulation of reactive oxygen species (ROS) can lead to the downregulation of NIS in DTC cells (78), suggesting that diabetes may promote the RAIR-DTC development.

In addition to diabetes mellitus, anti-diabetic medications such as Metformin, an AMPK agonist approved by the U.S. Food and Drug Administration (FDA), may have varying effects patients. While some studies indicate that metformin reduces tumor volume in diabetic patients with RAIR-DTC (83), others report a decreases in NIS expression and iodine uptake *in vitro (* 84). Consequently, the use of Metformin in diabetic patients with RAIR-DTC may yield antagonistic effects through AMPK activation.

#### 2.5.2 Myeloid leukemia and RAIR-DTC

Evidence suggests that prolonged RAI therapy exceeding 100 mCi may increase the risk of acute or chronic myeloid leukemia (AML or CML) in DTC patients, particularly males. This risk is potentially linked to ROS induced oxidative stress and subsequent DNA damage (71, 74, 85). Supporting this mechanism, Rajeshwari et al. observed elevated lipid peroxidation and protein oxidation in AML (N=30) and CML (N=30) patients compared to controls (N=13) (86). Similarly, Zhou et al. and Pascu et al. emphasized oxidative stress’s role in the etiology and treatment of AML and CML (87, 88). Furthermore, Cazarin et al. noted that ROS can alter the expression and function of the NIS in thyrocytes and TC cells (89).

Nilotinib, an FDA-approved BCR-ABL inhibitor for CML, enhances TC sensitivity to RAI by inducing autophagy through PI3K/Akt pathway inhibition (90, 91). However, it also activates AMPK, which promotes NIS degradation. This reduces RAI uptake and can induce RAIR (92, 93).

#### 2.5.3 Blood pressure disorder and RAIR-DTC

Yu et al. found that altered thyroid hormone (TH) sensitivity—specifically reduced central sensitivity and increased peripheral sensitivity—is associated with a heightened risk of hypertension in patients with coronary heart disease (N=34,310). Logistic regression analysis revealed an inverse relationship between central thyroid resistance (reflected by the TSH index) and hypertension risk, alongside a positive correlation between the FT3/FT4 ratio (indicating peripheral sensitivity) and hypertension risk (94). Separately, MKIs like sorafenib and Lenvatinib, which inhibit the VEGF axis, are associated with hypertension in advanced DTC (72, 95). Furthermore, although less common, Kitamura et al. observed that sternal or mediastinal lymph node metastases in metastatic TC can compress the vena cava, causing hypotension and sudden death (75). Collectively, these findings suggest that blood pressure dysregulation may influence the incidence or progression of RAIR-DTC.

In the management of blood pressure disorders in patients with DTC, anti-hypertensive medications such as Valsartan, Captopril, and Nifedipine, along with anti-hypotensive drug Etilefrine, which is known for its activation of the AMPK pathway, are used (96–99). Consequently, these medications may influence the expression of the NIS and RAI uptake through AMPK activation, potentially impacting the development and progression of RAIR-DTC.

### 2.6 Clinical implication

We recommend that patients with DTC undergo regular monitoring of RAIR-DTC related gene mutations and serum biomarker expression levels following initial treatment to prevent disease progression. Although clinical evidence has not confirmed that diabetes, myeloid leukemia (ML), blood pressure disorders, or treatments such as metformin, nilotinib, etilefrine, and valsartan directly promote RAIR-DTC or worsen its prognosis, research suggests a potential link via AMPK pathway activation, which may contribute to adverse outcomes in DTC (80, 84, 93, 96, 97, 100). Given the retrospective nature of available studies and uncertain causality, caution is advised when prescribing these medications to DTC patients with relevant comorbidities.

## 3 Molecular features in RAIR-DTC

**Figure 2:**
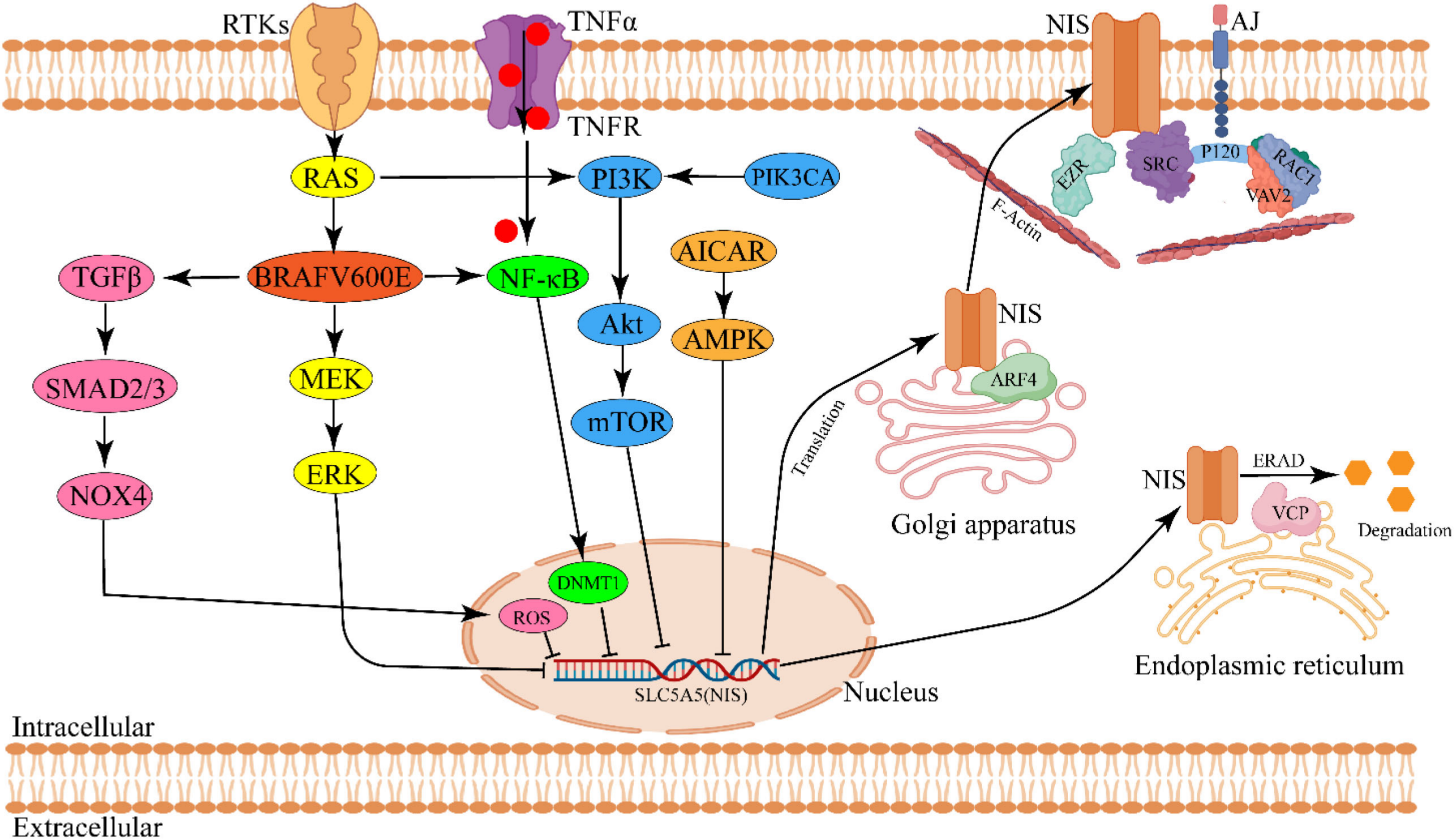
Schematic diagram of molecular pathogenesis in modulating NIS expression and activity in RAIR-DTC. Aberrant activation of signaling pathway derived by gene mutation (RAS/BRAF) is responsible for regulating NIS expression. NIS membrane targeting and glycosylation modulate NIS activity. ARF4, ADP-ribosylation factor 4; AICAR, AMPK agonist 5-aminoimidazole-4-carboxamide-ribonucleoside; DNMT1, DNA methyltransferase1; ERAD, Endoplasmic-reticulum-associated protein degradation; EZR, EZRIN; VCP, Valosin-containing protein. The image was created with BioRender.com.

Existing evidence indicates that the core molecular pathogenesis of RAIR-DTC involves downregulation or functional impairment of the sodium-iodide symporter (NIS), which prevents effective RAI uptake. This NIS dysfunction results from dysregulated signaling pathways driven by genetic alterations, including RAS mutations, BRAF^V600E^ mutations, TERT promoter mutations, RET/NTRK fusions, and PIK3CA mutations) (103). Here, we elaborate on how these abnormally activated signaling pathways impact NIS expression or activity and consequently reduce RAI efficacy (46, 48, 52) (**Figure 2**).

### 3.1 Decrease in NIS expression

#### 3.1.1 MAPK signaling pathway

Mitogen-activated protein kinase (MAPK) cascades in mammalian cells consist of three major families: extracellular signal-regulated kinases (ERKs), c-Jun N-terminal kinases (JNKs), and p38/stress-activated protein kinases (SAPKs) (106). The MAPK pathway, a downstream branch of BRAF signaling, significantly influences the expression of key genes involved in thyroid hormone biosynthesis, including NIS, TPO, and TG, and plays a crucial role in the progression of RAIR-DTC (107, 108). Liu et al. demonstrated that inhibiting MEK or silencing the BRAF^V600E^ mutation in PCCL3 cells (a rat thyroid cell line) restored the expression of iodide-metabolizing genes (109). Furthermore, BRAF^V600E^ induced deacetylation of nucleotides −297/−107 in the rat NIS promoter and −692/−370 in the human NIS promoter contributes to NIS gene silencing (110). Overexpression of miR-20b has been shown to reduce cell viability and migration in human TC cells and to suppress tumor growth in xenografts by directly targeting SOS1 and ERK2, thereby inhibiting the MAPK/ERK pathway (111). Similarly, Shen et al. reported that miR-106a overexpression decreased NIS expression and RAI uptake in TC cells by directly targeting retinoic acid receptor beta (RARB) and activating the MAPK pathway *in vitro (* 112). Overall, BRAF^V600E^ mediated hyperactivation of the MAPK pathway plays a key role in suppressing NIS expression.

#### 3.1.2 PI3K/Akt signaling pathway

Besides regulating cell growth, transcription, and translation, the phosphoinositide 3-kinase (PI3K) pathway plays a significant role in the pathogenesis of RAIR-DTC (113). Bibian et al. demonstrated that IGF1-mediated activation of PI3K markedly inhibited TSH-dependent NIS transcription in FRTL5 cells, a rat thyroid cell line (114). Similarly, Kogai et al. showed that the PI3K inhibitor LY294002 significantly increased iodide uptake and enhanced PAX8 expression in PCCL3 cells by stimulating NIS post-translational regulation (115). Pharmacological inhibition of PI3K led to approximately a 3.5-fold increase in exogenous NIS expression and iodine uptake in BHP 2–7 cells, which harbor the RET/PTC1 rearrangement (115). Liu et al. found that inhibition of Akt using Akti-1/2 in PCCL3 cells did not increase NIS protein expression but significantly reduced iodide efflux and enhanced the affinity between iodine and NIS, thereby promoting RAI uptake (116). Rapamycin, an mTOR inhibitor, improved iodine uptake and reduced cell viability in PCCL3 cells, despite not elevating NIS protein levels. Its synthetic analog, everolimus, similarly enhanced thyroid iodine uptake *in vivo *(117). Moreover, Plantinga et al. reported that rapamycin increased both mRNA and protein levels of human NIS (hNIS) and RAI uptake capacity in TC cells by inhibiting mTOR via transcriptional upregulation of TTF1 expression (118). Additionally, Chen et al. found that overexpression of small nucleolar RNA host gene 7 upregulated dipeptidyl peptidase 4 (DPP4), promoting TC cell proliferation and I^131^ resistance through activation of the PI3K/Akt pathway (119). In summary, these findings support the conclusion that aberrant activation of the PI3K/Akt/mTOR pathway reduces NIS mediated radioiodine accumulation in the thyroid gland.

#### 3.1.3 AMPK signaling pathway

The AMP-activated protein kinase (AMPK) signaling pathway is activated in response to cellular energy depletion and is notably upregulated in PTCs, contributing to TC progression (92). The AMPK agonist 5-aminoimidazole-4-carboxamide ribonucleoside (AICAR) significantly reduced NIS mRNA and protein expression in PCCL3 cells. However, this effect was reversed by TSH-induced activation of the cyclic adenosine monophosphate (cAMP)/protein kinase A pathway or treatment with bafilomycin A1, both of which inhibited AMPK signaling (92, 93). Gonçalves et al. demonstrated that rutin increased NIS mRNA and protein expression and reduced RAI efflux both *in vitro* and *in vivo* by inhibiting the AMPK pathway and ROS generation (120, 121). Additionally, Compound C, an AMPK inhibitor, enhanced NIS expression by promoting activation of the cyclic AMP response element (CRE) in the NIS promoter, through suppression of AMPK signaling (84). In summary, aberrant activation of the AMPK pathway can downregulate RAI uptake in both non-neoplastic thyroid follicular cells and TC cells.

#### 3.1.4 Other signaling pathway

Activation of the transforming growth factor-beta (TGF-β) and nuclear factor kappa-light-chain-enhancer of activated B cells (NF-κB) signaling pathways also negatively regulates NIS expression and RAI uptake in the thyroid gland. Further details are available in reference (104).

### 3.2 Impairment of NIS function

In addition to adequate overall NIS expression, proper localization, dimerization, and glycosylation of NIS are also essential for enhancing the efficacy of RAI therapy (105, 122). Faria et al. demonstrated that P120 catenin, a component of adherens junctions (AJs), recruits SRC and RAC1 kinases. SRC phosphorylates VAV2, which in turn activates RAC1, promoting NIS retention on the plasma membrane (PM) (105, 122). Fletcher et al. further showed that ADP-ribosylation factor 4 (ARF4) increases NIS presence on the PM by facilitating its transport from the Golgi apparatus, while valosin-containing protein (VCP) mediates NIS degradation (123). Additionally, Thompson et al. reported that point mutations in NIS residues (e.g., Y242 and T243) impair RAI uptake by disrupting NIS dimerization (124). Regarding glycosylation, Chung et al. found that cAMP enhances hNIS expression, membrane localization, and RAI uptake by promoting NIS glycosylation in HeLa cells and xenograft models. These effects were reversed by tunicamycin, which inhibited NIS glycosylation *in vitro* (125). Collectively, disruptions in PM localization, dimerization, or glycosylation impair NIS function and reduce RAI uptake (**Figure 2**).

## 4 Current clinical treatments and clinical trials

**Figure 3:**
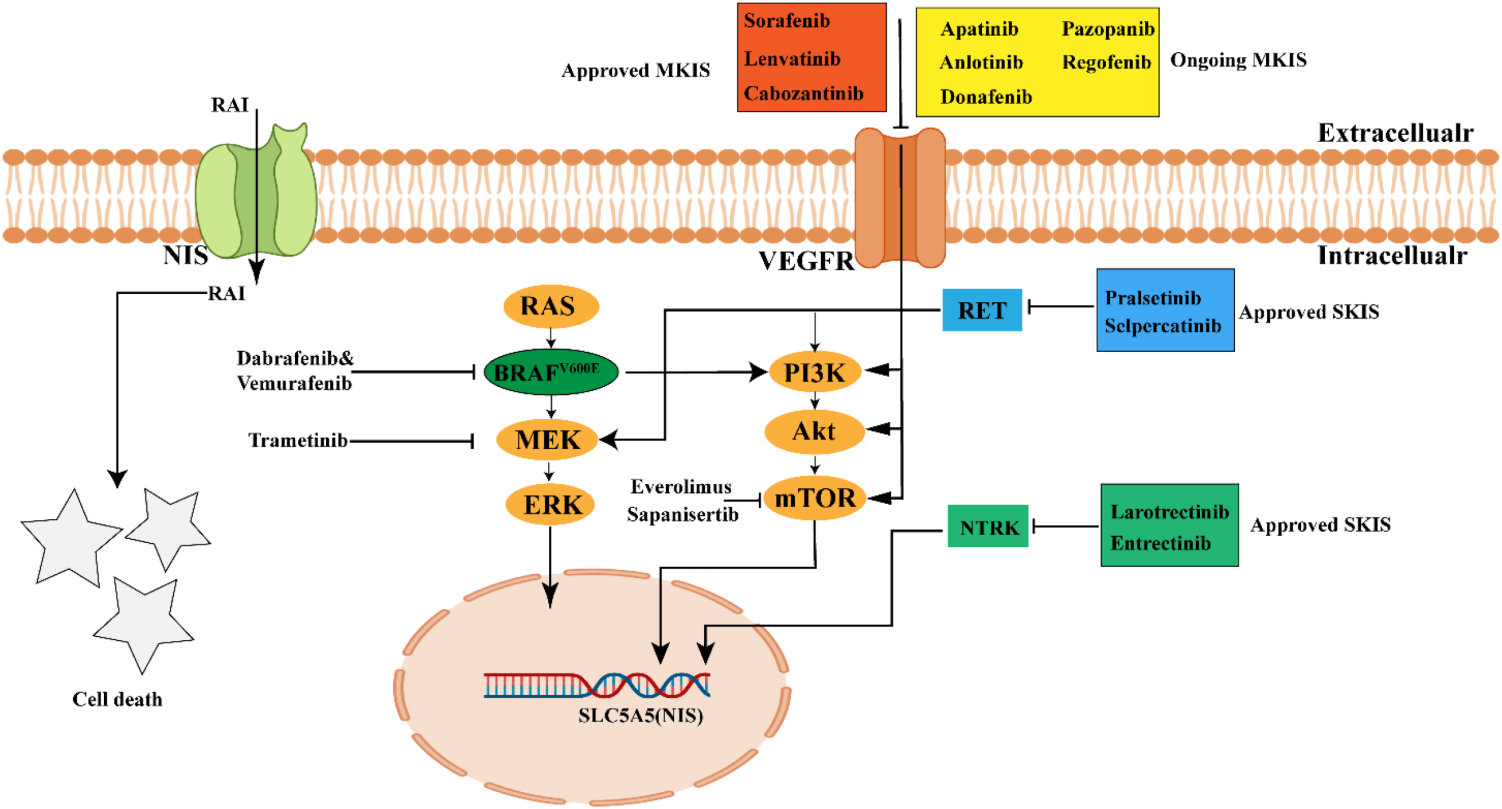
Current treatment drugs in RAIR-DTCs. MKIs (such as Lenvatinib, Sorafenib, Cabozantinib) and SKIs (such as Pralsetinib, Selpercatinib, Larotrectinib and Entrectinib) have been approved by FDA to treat RAIR-DTCs. Other kinase inhibitors are ongoing in Clinical trial. MKIs, muliti-kinase inhibitors; SKIs, selective kinase inhibitors; The picture was created with BioRender.com.

Given the heterogeneity of RAIR-DTC, a range of treatment strategies is employed in clinical practice. Asymptomatic and indolent cases are typically managed with suppressive levothyroxine therapy, while symptomatic, progressive oligometastatic RAIR-DTC may require surgical intervention, external beam radiotherapy, or thermal ablation (10). In contrast, symptomatic, rapidly progressive, inoperable locally advanced or widely metastatic RAIR-DTC presents significant clinical challenges and is often primarily treated with multi-kinase inhibitors (MKIs), selective kinase inhibitors (SKIs), or redifferentiation therapy (126, 127). Due to its complexity, this aggressive subtype has become a primary focus of our clinical management efforts. Unlike cytotoxic chemotherapy, these newer targeted therapies have significantly prolonged PFS in patients with RAIR-DTC (**Table 5**). However, improvements in OS have been limited, with only Apatinib and Lenvatinib (in patients over 65) showing OS benefits. In this review, we retrospectively analyze and synthesize the efficacy and safety profiles of targeted and redifferentiation therapies, both approved agents and those under clinical investigation, used as monotherapy or in combination, as summarized below (**Figure 3**).

**Table 5:** Summary of different MKIs in RAIR-DTCs that are either approved or undergoing clinical trials.

NCT Num.	No. of subjects	Drug	Target	Phase	FDA approval	PFS&ORR	Primary AEs	Ref.
NCT00984282	N=417	Sorafenib	VEGFR 1–3, PDGFRA, RET, FLT3, c-KIT and RAF (including CRAF, BRAF ^V600E^)	/	Yes	PFS: Sorafenib(10.8 months) vs. placebo(5.8 months)	Hand-foot skin reaction (76.3%), diarrhea (68.6%), alopecia (67.1%), and rash or desquamation (50.2%)	(72)
NCT01321554	N=392	Lenvatinib	VEGFR1-3, FGFR 1-4, PDGFR-α, RET and KIT	/	Yes	PFS: Lenvatinib (18.3 months) vs. placebo (3.6 months); ORR: Lenvatinib (64.8%) vs. placebo (1.5%)	Hypertension (67.8%), diarrhea (59.4%), fatigue or asthenia (59.0%), decreased appetite (50.2%), weight-loss (46.4%), and nausea (41.0%)	(95)
NCT03690388	N=187	Cabozantinib	MET, RET, AXL, VEGFR1-3, FLT3, NTRK and c-KIT	/	Yes	ORR: Cabozantinib (15%) vs. placebo (0%)	Palmar-plantar erythrodysaesthesia (10%), hypertension (9%), and fatigue (8%).	(142)
NCT03048877	N=92	Apatinib	VEGFR2, PDGF-β, C-kit, BRAF^V600E^	phase III	No	ORR: Apatinib(54.3%) vs placebo(2.2%);DCR: Apatinib(95.7%) vs placebo(58.7%);median PFS: Apatinib (22.2 months) vs placebo(4.5 months)	Hypertension (34.8%), hand-foot syndrome (17.4%), proteinuria (15.2%), and diarrhea (15.2%)	(15)
NCT02586337	N=113	Anlotinib	VEGFR, PDGFR, FGFR, and c-KIT	phase II	No	mPFS: Anlotinib (40.5 months)vs.placebo (8.4 months)	Palmar-plantar erythrodysesthesia syndrome (74%), hypertension (84%), and proteinuria (65%).	(143)
	N=191	Donafenib	VEGFR1-3, PDGFR, RAF	phase III	No	mPFS: Donafenib(12.9 months) vs Placebo(6.4months); ORR: Donafentib(23.3%) vs Placebo(1.7%)	Hypertension (13.3%) and hand-foot syndrome (12.5%)	(144)
NCT 00625846	N=60	Pazopanib	VEGFR1-3, PDGFR-α PDGFR-β,c-Kit, FGFR1,FGFR3	Phase II	No	mPFS:11.4 months, mOS:2.6 years	Hypertension, Diarrhea	(145)
NCT02657551	N=9	Regorafenib	VEGFR1-3, RET, FGFR, DDR2	Phase II	No	ORR was 11.1%, mPFS:11 months, mOS: 20.1 months	Diarrhea, hypophosphatemia, hypertension	(146)

OS, Overall survival; PFS, Progressively free survival; mPFS, CRR, Complete response rate; PRR, Partial response rate; ORR, Objective response rate.

### 4.1 Targeted therapy

Targeted therapy for RAIR-DTC primarily relies on the use of MKIs and SKIs to achieve therapeutic efficacy (126). The MKIs function by inhibiting a range of receptors and kinases, including the vascular endothelial growth factor receptor (VEGFR), platelet-derived growth factor receptor (PDGFR), epidermal growth factor receptor (EGFR), human epidermal growth factor receptor 2 (HER2), and fibroblast growth factor receptor (FGFR), thereby suppressing tumor cell proliferation and growth (128). Notably, Sorafenib, Lenvatinib, and Cabozantinib have been approved by the FDA for the treatment of RAIR-DTC (129–131), while other MKIs are still under clinical investigation. Regarding SKIs, the FDA has approved RET inhibitors (Selpercatinib and Pralsetinib) and NTRK fusion inhibitors (Larotrectinib and Entrectinib) for RAIR-DTC patients with RET or NTRK alterations (132–135). In this section, we highlight the clinical progress of FDA approved MKIs and SKIs in improving RAIR-DTC prognosis and summarize other MKIs currently in clinical trials (**Tables 5**, **6**).

**Table 6:** Summary of different selective kinase inhibitors (SKIs) in RAIR-DTC that are either approved or undergoing clinical trials.

NCT Num.	No. of subjects	Drug	Target	Phase	FDA approval	PFS&ORR	Primary AEs	Ref.
NCT03037385	N=22	Pralsetinib	RET	/	Yes	ORR:90.9%, CRR:13.6%, PRR:77.3%	Increased aspartate aminotransferase, anemia, and hypertension	(131)
NCT03157128	N=19	Selpercatinib	RET	/	Yes	ORR:79%, Rate of duration of response>6 months is 87%	Hepatotoxicity, hypertension and embryo-fetal toxicity.	(134)
/	N=355	Larotrectinib	NTRK	/	Yes	ORR:58%	Constipation, Dizziness, Fatigue	(132)
/	N=54	Entrectinib	NTRK	/	Yes	ORR:57%, Rate of duration of response>6 months is 68%	Pulmonary infections, weight gain, dyspnea	(133)
NCT02145143	N=12	Vemurafenib	BRAF ^V600E^	phase II	No	ORR: vemurafenib (60%%)	/	(147)
/	N=53	Dabrafenib &Trametinib	BRAF & MEK1/2	phase II	No	ORR: Dabrafenib+trametinib(48%) vs Dabrafenib(42%)	Skin and subcutaneous tissue disorders (17/26, 65%), fever (13/26, 50%),with dabrafenib alone and fever (16/27, 59%), nausea with dabrafenib + trametinib	(148)
NCT02143726	N=35	Everolimus	mTOR	Phase II	No	PFS: Sorafenib + Everolimus (24.7 months) vs Sorafentinib(10.9 months)	Cardiac arrest, tracheal obstruction	(23)
NCT02244463.	N=22	Sapanisertib	mTOR	phase I/II	No	PRR (4.5%), stable disease in 63.6% patients. mPFS was 7.8 months	Anorexia, nausea, diarrhea,	(149)

OS, Overall survival; PFS, Progressively free survival; mPFS, median Progressively free survival; CRR, Complete response rate; PRR, Partial response rate; ORR, Objective response rate.

#### 4.1.1 Approved MKIs

##### 4.1.1.1 Sorafenib and Lenvatinib

Sorafenib, a multi-targeted kinase inhibitor, exhibits activity against *RET*, *CRAF*, *BRAF* (including both wild-type and V600E mutant), *VEGFR1-3*, *FLT3*, and *c-KIT*. In a multicenter, randomized, double-blind, placebo-controlled, phase 3 clinical trial (N=417, NCT00984282), Sorafenib significantly prolonged the PFS of RAIR-DTCs than placebo (10.8 vs. 5.8 months) (72). Consequently, Sorafenib became the first MKI approved by the FDA for the treatment of RAIR-DTC in both first-line and second-line settings (136). Similarly, Lenvatinib, another oral MKI targeting *VEGFR1-3*, *FGFR1-4*, *PDGFRα*, and *RET*, markedly extended the PFS in RAIR-DTCs than placebo (18.3 vs. 3.6 months) in a randomized, double-blind, multicenter phase 3 clinical trial(NCT01321554) (95). Subsequently, Lenvatinib also received FDA approval for use as a first- or second-line therapy in the RAIR-DTC treatment (129). Notably, a sub-analysis evaluating age-related efficacy and safety of Lenvatinib indicated a significant OS advantage over placebo in patients aged over 65 years old(*P*=0.02) (14). As for it, a phase 3 clinical trial conducted in the Chinese population demonstrated that Lenvatinib significantly improved PFS (23.9 months vs. 3.7 months) and objective response rate(ORR)(59% vs.24%) in RAIR-DTC compared to placebo, accompanied with several AEs similar to those observed in Schlumberger’s clinical studies (N=151, NCT02966093) (137).

##### 4.1.1.2 Cabozantinib

Cabozantinib was developed to overcome resistance to VEGFR inhibitors by targeting the MET pathway (138), and it significantly prolonged PFS compared with control (11.0 months vs. 1.9 months) in a randomized, double-blind, placebo-controlled phase 3 trial (NCT03690388). At present, Cabozantinib is approved for the treatment of adult patients with locally advanced or metastatic DTC who have progressed after receiving VEGFR targeted therapy. It is also indicated a second-line treatment option for RAIR-DTCs after the failure of first-line treatment, such as Lenvatinib.

#### 4.1.2 Unapproved MKIs

##### 4.1.2.1 Apatinib

Apatinib is an oral inhibitor that targets *VEGFR2*, *PDGFR-β*, *C-kit*, and *BRAF^V600E^*. In a phase 3 randomized clinical trial involving 92 patients with RAIR-DTC, Apatinib significantly prolonged PFS compared to placebo (22.2 vs. 4.5 months). The ORR in the Apatinib group was 54.3%, with a disease control rate (DCR) of 95.7%, compared to an ORR of 2.2% and a DCR of 58.7% in the placebo group. The most common grade ≥3 AE was hypertension, occurring in 34.8% of participants (15).

##### 4.1.2.2 Anlotinib

As a MKIs, Anlotinib targets *VEGFR*, *PDGFR*, *FGFR*, and *c-KIT*. In a randomized, double-blind, multicenter phase 2 trial of Anlotinib in locally advanced or metastatic RAIR-DTC (NCT02586337), the median PFS was 40.54 months in Anlotinib and 8.38 months in placebo. The ORR was 59.21% in Anlotinib, in addition, significant DCR benefit was observed in Anlotinib treatment (97.37% vs. 78.38%). The most common AEs were hypertension (84.21%) and hypertriglyceridemia (68.42%) (139). Although the Anlotinib is not approved by the FDA, but it is approved by the China National Medical Products Administration (NMPA) for the indication of RAIR-DTC, based on its promising efficacy.

##### 4.1.2.3 Donafenib

Donafenib is a novel oral MKI that targets *VEGFR1-3*, *PDGFR*, and *RAF*. In a multicenter, randomized, double-blind phase 3 trial, Donafenib demonstrated a prolonged median PFS compared to placebo (12.9 vs. 6.4 months) in Chinese RAIR-DTCs. Additionally, the Donafenib group exhibited improved ORR of 23.3% compared to 1.7% for placebo (*P* = 0.0002) and DCR of 93.3% versus 79.3% for placebo (*P* = 0.0044). The most common grade≥3 treatment-related AEs associated with Donafenib included hypertension (13.3%) and hand-foot syndrome (12.5%). 42.2% of patients required dose reduction or interruption, while 6.3% experienced treatment discontinuation (140). Based on the safety and efficacy data of Donafenib in clinical treatments, it has been approved by the NMPA for the treatment of RAIR-DTC.

##### 4.1.2.4 Pazopanib

Pazopanib is an oral inhibitor of *VEGFR1-3*, *PDGFR-α/β*, *c-Kit*, and *FGFR1/3*. In an international phase 2 study of progressive and metastatic thyroglobulin antibody negative RAIR-DTCs (NCT00625846), the median PFS and OS of Pazopanib group were estimated to be 11.4 months and 2.6 years from start of study therapy initiation, respectively. Common AEs included one death (thromboembolic) deemed possibly associated with pazopanib (141).

##### 4.1.2.5 Regorafenib

Regorafenib is an oral inhibitor of *VEGFR1-3*, *RET*, *FGFR* and *DDR2*. In a phase 2 trial of RAIR-DTCs (NCT02657551), the ORR of Regorafenib was 11.1%, median PFS and OS were 11.0 and 20.1 months respectively. As for AEs, it mainly includes diarrhea, hypophosphatemia and hypertension and there were no treatment-related deaths (142). Overall, the clinical efficacy of Regorafenib is not ideal.

#### 4.1.3 Approved selective kinase inhibitors

##### 4.1.3.1 RET inhibitors

Currently, the FDA has approved RET inhibitors (Selpercatinib and Pralsetinib) for adult and pediatric patients aged 12 years and older with advanced or metastatic RET fusion-positive TC who require systemic therapy and are classified as RAIR. Subbiah et al. conducted a study demonstrating that the ORR of Pralsetinib in patients with RET fusion-positive thyroid cancer who had received prior systemic treatment was 90.9% (N=22), with several AEs reported, including increased aspartate aminotransferase, anemia, and hypertension (132). The efficacy of Selpercatinib was evaluated in 27 RAIR-DTCs with RET fusion-positive and ORR was 85%, common AEs include diarrhea, hypertension and fatigue (135).

##### 4.1.3.2 NTRK fusion inhibitors

Moreover, NTRK inhibitors, namely Larotrectinib and Entrectinib, have been approved for patients harboring NTRK1–3 fusion mutations in RAIR-DTC. Waguespack et al. analyzed the ORR of Larotrectinib, which was found to be 71%(N=28) in NTRK fusion positive TCs (143). Notably, the most common AEs associated with Larotrectinib include fatigue, cough, and constipation. The ORR of Entrectinib in TCs with NTRK fusion positivity was reported at 20% (N=5), with a duration of response (DOR) of 7.9 months (134). The most frequently observed AEs for Entrectinib included pulmonary infections, weight gain, and dyspnea.

### 4.2 Redifferentiation therapy

Due to dedifferentiation, the functional expression of the NIS is diminished in RAIR-DTC, consequently reducing the uptake and efficacy of RAI. Redifferentiation therapy aims to restore RAI uptake capacity by enhancing NIS expression. Current, clinical research has demonstrated the potential of BRAF inhibitors (Dabrafenib and Vemurafenib) (145, 148), MAPK kinase (MEK) inhibitors (Trametinib) (149) and mTOR inhibitors (Everolimus and Sapanisertib) (24, 147) to enhance NIS expression and improve iodine uptake. Notably, Leboulleux et al. conducted a phase 2 clinical trial, showing that the combination of Dabrafenib and Trametinib for redifferentiation therapy significantly improved the efficacy of I^131^ uptake in BRAF^V600E^ mutant RAIR-DTCs, with 38% of patients achieving partial remission and 52% presenting with stable disease (25). Moreover, Sherman et al. conducted a phase 2 clinical trial, demonstrating that the combination of Sorafenib and Everolimus for redifferentiation therapy significantly prolonged the PFS in RAIR-DTCs than Sorafenib alone(24.7 vs 10.7 months) (24). Although these inhibitors have demonstrated promising efficacy in clinical trials, they have not yet been approved by the FDA and require further clinical trial evaluation.

These clinical investigations demonstrate that target and redifferentiation therapies can improve the living conditions of patients in RAIR-DTCs, but the high cost and serious AEs have not yet reached the expectations of patients. The future drug development should be closely combined with the molecular pathogenesis, namely the development of specific and effective drugs against RAIR-DTC should be a mainstream direction.

## 5 Conclusions

In recent decades, there has been a significant improvement in treatment outcomes and prognoses for DTCs. Nonetheless, approximately 10% of patients with DTC still experience recurrence, metastasis, and resistance to RAI, leading to a markedly poor prognosis. Despite considerable advancements in elucidating the molecular mechanisms and developing targeted therapies for RAIR-DTC, SKIs or MKIs including those approved or currently undergoing clinical trial have demonstrated improvement in PFS. However, most targeted therapies have not demonstrated substantial enhancements in in OS, with the notable exceptions of Lenvatinib and Apatinib, which are associated with numerous AEs.

To reduce the incidence of RAIR-DTC and improve its treatment prognosis, we first summarized high-risk factors of RAIR-DTC in this review. Through detecting genetic mutations (e.g., RAS/BRAF/TERT promoter), clinical pathological features (e.g., ETE, and distant metastasis), and early monitoring biomarkers(e.g., Cyfra21.1 and TG) could aids in the early detection or prediction of RAIR and timely clinical intervention (34, 47, 56, 58, 63). Importantly, we have discussed several potential inducing factors stemming from comorbidities (e.g., diabetes, ML, and blood pressure disorders) and unsuitable medications (e.g., Metformin, Nilotinib, Etilefrine, etc.) that could activate the AMPK pathway, thereby inducing RAIR (84, 96, 97, 100–102). Subsequent, we reviewed the molecular mechanisms by which the abnormal activation of signaling pathways, driven by genetic mutations, leads to impaired expression and function of NIS in RAIR-DTC. Concurrently, we summarized the safety and efficacy of KIs that are currently approved by the FDA for RAIR-DTC, as well as those undergoing clinical trials. Based on the results of ongoing clinical trials, although existing drugs demonstrate certain efficacy, they are frequently associated with severe AEs. Future efforts should focus on further exploring the molecular mechanisms, optimizing existing drugs, and developing new RAIR-DTC treatments that are safe, effective, and specific.
